# Ureteroilleal anastomosis in orthotopic ileal neobladders: Does technique matter?

**Published:** 2008

**Authors:** Parag Gupta, K. Muruganandham, Aneesh Srivastava

**Affiliations:** Department of Urology and Renal transplantation, SGPGIMS, Lucknow, UP, India. E-mail: anees772000@yahoo.com

## SUMMARY

In a randomized prospective trial of 60 patients undergoing orthotopic ileal neobladder, Atallah and Mohamed *et al.* assessed the pros and cons of refluxing and antirefluxing ureteroileal anastomosis. Patients underwent direct anastomosis of ureter on one side and antireflux serous lined extramural tunnel on the contralateral side.[1] Glomerular filtration rate (GFR) wa^~^ 15 patients. One patient had prolonged leakage from a refluxing uretero-ileal anastomosis, which was managed with temporary percutaneous nephrostomy. Early anastomotic strictures developed in six patients, one with direct and five with antirefluxing technique. Strictures were more common on left than right side (4 vs. 2). Out of these six patients, three were treated with double J-stenting and transurethral endoureterotomy while other three required open ureteric reimplantation. The mean presurgery GFR decreased from 48.6 to 31.8 ml/min after managing these stricture units (*P*= 0.01).

## COMMENTS

This is a well-designed, prospective, randomized study comparing antireflux and direct uretero-ileal anastomosis in the same patient with an orthotopic ileal neobladder.

The orthotopic bladder substitute has evolved into the most ideal form of urinary diversion available today and it may be considered the gold standard. Controversy exists regarding the need to incorporate an antireflux mechanism in patients undergoing an orthotopic form of urinary diversion. Even though there are many reports in the literature addressing this issue, these are all retrospective studies with their own limitations.

In the present study, the incidence of ureteroileal stricture was five times higher with antirefluxing anastomosis compared to direct method. This is consistent with the reported incidence of anastomotic strictures of 7.8-20.4% after antirefluxing procedures vs. 1.4-3.6% after direct methods.[2–4] No significant difference in GFR was detected between the methods of uretero-ileal implantation during short-term follow-up. Incidence of asymptomatic bacteriuria also declines steadily with time so as such will not make much difference in GFR later on.[5]

Although the incidence of asymptomatic bacteriuria is more common with the refluxing ureteroileal anastamosis, it gradually decreases over time and it does not affect the renal function. Moreover, the stricture rates are found to be more common with the antirefluxing anastamosis. The results from this well-conducted study support refluxing rather than antirefluxing anastamosis in orthotopic neobladder reconstruction. However, studies with longer follow-up may require to support or contradict this finding.

## References

[1] Shaaban AA, Abdel-Latif M, Mosbah A, Gad H, Eraky I, Ali-El-Dein B (2006). A randomized study comparing an antireflux system with a direct ureteric anastomosis in patients with orthotopic ileal neobladders. BJU Int.

[2] Pantuck AJ, Han KR, Perrotti M, Weiss RE, Cummings KB (2000). Ureteroenteric anastomosis in continent urinary diversion: Long-term results and complications of direct versus nonrefluxing techniques. J Urol.

[3] Gschwend JE, Volkmer BG, Liske P, Kleinschidt K, Hautmann RE (2005). Type of ureteral anastomosis in orthotopic ileal neobladder: Impact on the rate of hydronephrosis and renal function. J Urol.

[4] Roth S, Van Ahlen H, Sejonow A, Oberpenning F, Hertle L (1997). Does the success of ureterointestinal implantation in orthotopic bladder substitution depend more on surgeon level of experience or choice of technique?. J Urol.

[5] Abdel-Latif M, Mosbah A, El-Bahnasawy MS, El-Sawy E, Shaaban AA (2005). Asymptomatic bacteriuria in men with orthotopic ileal neobladders: Possible relationship to nocturnal enuresis. BJU Int.

